# Cited2 Is an Essential Regulator of Adult Hematopoietic Stem Cells

**DOI:** 10.1016/j.stem.2009.11.001

**Published:** 2009-12-04

**Authors:** Kamil R. Kranc, Hein Schepers, Neil P. Rodrigues, Simon Bamforth, Ellen Villadsen, Helen Ferry, Tiphaine Bouriez-Jones, Mikael Sigvardsson, Shoumo Bhattacharya, Sten Eirik Jacobsen, Tariq Enver

**Affiliations:** 1 MRC Molecular Haematology Unit, Weatherall Institute of Molecular Medicine, John Radcliffe Hospital, University of Oxford, OX3 9DS Oxford, UK; 2 Haematopoietic Stem Cell Laboratory, Weatherall Institute of Molecular Medicine, John Radcliffe Hospital, University of Oxford, OX3 9DS Oxford, UK; 3 Institute of Human Genetics, Newcastle University, International Centre for Life, Central Parkway, Newcastle upon Tyne, NE1 3BZ, UK; 4 Department for Clinical and Experimental Research, Linköping University, 58185 Linköping, Sweden; 5 Wellcome Trust Centre for Human Genetics, University of Oxford, OX3 7BN Oxford, UK

**Keywords:** STEMCELL

## Abstract

The regulatory pathways necessary for the maintenance of adult hematopoietic stem cells (HSCs) remain poorly defined. By using loss-of-function approaches, we report a selective and cell-autonomous requirement for the p300/CBP-binding transcriptional coactivator Cited2 in adult HSC maintenance. Conditional deletion of *Cited2* in the adult mouse results in loss of HSCs causing multilineage bone marrow failure and increased lethality. In contrast, conditional ablation of *Cited2* after lineage specification in lymphoid and myeloid lineages has no impact on the maintenance of these lineages. Additional deletion of *Ink4a/Arf* (encoding p16^Ink4a^ and p19^Arf^) or *Trp53* (encoding p53, a downstream target of p19^Arf^) in a *Cited2*-deficient background restores HSC functionality and rescues mice from bone marrow failure. Furthermore, we show that the critical role of *Cited2* in primitive hematopoietic cells is conserved in humans. Taken together, our studies provide genetic evidence that *Cited2* selectively maintains adult HSC functions, at least in part, via *Ink4a/Arf* and *Trp53*.

## Introduction

Adult hematopoiesis depends on rare multipotent bone marrow (BM)-resident hematopoietic stem cells (HSCs) (Orkin and Zon, 2008). HSCs may remain quiescent, self-renew, undergo apoptosis, or differentiate into multiple blood lineages. Tight regulation of these fates is essential to maintain the adult HSC pool, and studies in mice have revealed some of the key regulators of HSC maintenance. To identify novel regulators of adult HSC maintenance, we and others employed comparative global gene expression approaches. These studies identified the p300/CBP-binding transcriptional coactivator Cited2 as a candidate regulator of adult HSCs (Gomes et al., 2002; Mansson et al., 2007; Zhong et al., 2005), but functional validation remains to be performed.

*CITED2* mutations are found in patients with congenital heart disease (Sperling et al., 2005), lending clinical significance in trying to understand *CITED2* function. Cited2 physically interacts with the histone acetyltransferase p300/CBP (Bhattacharya et al., 1999), coactivates DNA-binding transcription factors (Bamforth et al., 2001; Chou et al., 2006; Glenn and Maurer, 1999; Tien et al., 2004), and represses HIF-1-mediated transcription (Bhattacharya et al., 1999). *Cited2* has oncogenic properties (Sun et al., 1998) and controls proliferation of mouse embryonic fibroblasts (MEFs) via polycomb group genes *Bmi-1* and *Mel18* and the tumor suppressor *Ink4a/Arf* (Kranc et al., 2003). *Cited2* deletion in mice is embryonic lethal, causing multiple developmental defects (Bamforth et al., 2001; Yin et al., 2002), including impaired fetal liver hematopoiesis (Chen et al., 2007). Severe fetal liver malformations (Qu et al., 2007) precluded defining a cell-autonomous role for *Cited2* in HSC function and hematopoiesis, although these findings suggest a potential role for *Cited2* in fetal HSC regulation. In this study, we use a conditional knockout strategy to establish a requirement for *Cited2* in adult HSCs. Further, we demonstrate a role for *CITED2* in human hematopoiesis by RNA interference in CD34^+^ cord blood (CB) cells.

## Results

### *Cited2* Is Essential for Sustaining Multilineage Hematopoiesis

**Figure 1:**
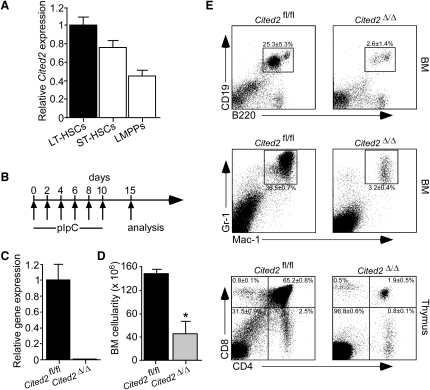
Conditional Deletion of *Cited2* Results in Multilineage Bone Marrow Failure (A) Relative expression of *Cited2* mRNA in LT-HSC, ST-HSC, and LMPP populations sorted from WT C57BL/6J mice. Data are mean ± SEM (n = 3). (B) *Cited2*^fl/fl^*Mx1-Cre* and *Cited2*^fl/fl^ mice received six injections of pIpC on alternate days and analyzed 5 days after the last injection. (C) Relative expression of *Cited2* mRNA in total BM cells from *Cited2*^Δ/Δ^ and control mice (mean ± SEM; n = 3). (D) Total number of BM nucleated cells obtained from two tibias and two femurs of *Cited2*^Δ/Δ^ and control mice. The results are presented as mean number of cells ± SD (n = 5). ^∗^p < 0.0001. (E) Top and middle: Frequencies of B-lymphoid and myeloid cells, respectively, in BM from *Cited2*^Δ/Δ^ and control mice. Bottom: FACS plot showing CD4 and CD8 staining in thymi from *Cited2*^Δ/Δ^ and control mice. Data are shown as mean frequency ± SD (n = 3).

*Cited2* expression analysis indicated that it is highly expressed in long-term HSCs (LT-HSCs; Lin^−^Sca-1^+^c-kit^+^(LSK)CD34^−^Flt3^−^ cells), less abundantly in short-term HSCs (ST-HSCs; LSKCD34^+^Flt3^−^ cells), and profoundly downregulated in lymphoid-primed multipotent progenitors (LMPPs; LSKCD34^+^Flt3^+^ cells) (Figure 1A). To investigate a functional requirement for *Cited2* in adult hematopoiesis, we generated *Cited2*^fl/fl^ *Mx1-Cre* conditional knockout mice (MacDonald et al., 2008), in which treatment with poly(I)-poly(C) (pIpC) induces efficient gene deletion in hematopoietic cells (Kuhn et al., 1995). We treated *Cited2*^fl/fl^ *Mx1-Cre* and *Cited2*^fl/fl^ mice with pIpC (Figure 1B) and refer to these as *Cited2*^Δ/Δ^ and *Cited2*^fl/fl^ mice, respectively. After Cre-mediated recombination, a *lacZ* expression cassette comes under the control of the endogenous *Cited2* promoter (MacDonald et al., 2008), and efficient gene deletion was demonstrated by abundant lacZ expression in *Cited2*^Δ/Δ^ BM cells (Figure S1A available online). Furthermore, *Cited2* mRNA was undetectable in *Cited2*^Δ/Δ^ BM cells (Figure 1C). Within 6 to 15 days after initiation of pIpC treatment, most *Cited2*^Δ/Δ^ mice became moribund and were sacrificed, in contrast to control mice, which survived normally (Figure S1B). BM analysis revealed severely reduced cellularity in *Cited2*^Δ/Δ^ mice (Figure 1D) and strikingly reduced frequencies of mature myeloid (Mac-1^+^Gr-1^+^) and B-lymphoid (CD19^+^B220^+^) cells in *Cited2*^Δ/Δ^ BM, as compared to control mice (Figure 1E). Conditional loss of *Cited2* also reduced T cell frequencies (Figure 1E). These data support an essential role for *Cited2* in sustaining adult multilineage hematopoiesis.

*Mx1-Cre* mediates gene deletion in both hematopoietic and nonhematopoietic tissues (Kuhn et al., 1995), so we assessed the contribution of *Cited2* deletion in nonhematopoietic tissues to morbidity. We transplanted wild-type (WT) BM cells into *Cited2*^fl/fl^ *Mx1-Cre* and *Cited2*^fl/fl^ mice, and 12 weeks after transplantation, recipients received pIpC. We observed no lethality in either cohort of mice (Figure S1C), indicating that BM failure in *Cited2*^Δ/Δ^ mice is the primary cause of mortality.

### *Cited2* Is Dispensable for the Maintenance of Committed Blood Lineages

**Figure 2:**
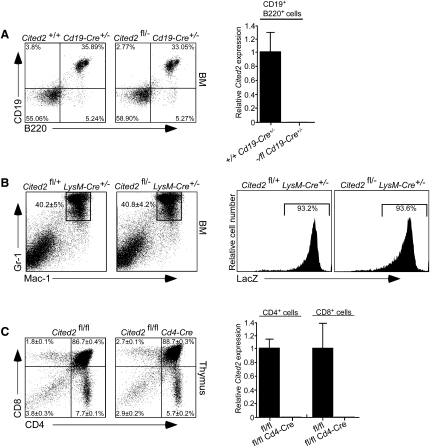
*Cited2* Is Dispensable for the Maintenance of Mature Lymphoid and Myeloid Lineages (A) Left: FACS plots showing CD19 and B220 staining of BM cells obtained from *Cited2*^+/+^*Cd19-Cre*^+/−^ and *Cited2*^fl/−^*Cd19-Cre*^+/−^ mice. Results from representative animals are shown (n = 3). Right: Relative expression of *Cited2* mRNA in CD19^+^B220^+^ cells sorted from peripheral blood (PB) of *Cited2*^+/+^*Cd19-Cre*^+/−^ and *Cited2*^fl/−^*Cd19-Cre*^+/−^ mice. Data are mean ± SEM (n = 3). (B) Left: FACS plot showing Mac-1 and Gr-1 staining of BM cells obtained from *Cited2*^fl/+^*LyzM-Cre*^+/−^ and *Cited2*^fl/−^*LyzM-Cre*^+/−^ mice. Data are shown as mean frequency ± SD (n = 3). Right: LacZ staining of BM Mac-1^+^Gr-1^+^ cells from *Cited2*^fl/+^*LyzM-Cre*^+/−^ and *Cited2*^fl/−^*LyzM-Cre*^+/−^ mice. (C) Left: FACS plots showing distribution of T cell subsets in the thymi of *Cited2*^fl/fl^ and *Cited2*^fl/fl^*Cd4-Cre* mice. Data are shown as mean frequency ± SD (n = 3). Right: Relative expression of *Cited2* mRNA in CD4^+^ and CD8^+^ cells sorted from PB of *Cited2*^fl/fl^ and *Cited2*^fl/fl^*Cd4-Cre* mice. Data are mean ± SEM (n = 3).

The multilineage defects observed in *Cited2*^Δ/Δ^ mice could reflect a requirement for *Cited2* in the maintenance of committed hematopoietic lineages. To test this hypothesis, we used *Cd19-Cre*, *LysM-Cre*, and *Cd4-Cre* strains to delete *Cited2* in B cell, myeloid, and T cell lineages, respectively. *Cd19-Cre* efficiently excised *Cited2* in CD19^+^B220^+^ cells but did not affect their frequency in the BM (Figure 2A). Likewise, efficient deletion of *Cited2* in the myeloid compartment led to lacZ expression in the majority of Mac-1^+^Gr-1^+^ cells, but did not alter the frequency of these cells in the BM (Figure 2B). *Cd4-Cre* efficiently excised *Cited2* in T cells but did not change their frequency in the thymus (Figure 2C). Therefore, *Cited2* is expendable for the maintenance of these committed lineages.

### *Cited2* Is Required for the Maintenance of Adult HSCs

**Figure 3:**
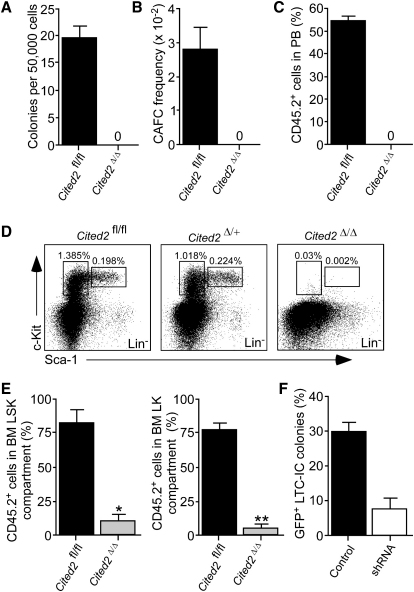
*Cited2* Maintains HSCs in a Cell-Autonomous Manner *Cited2*^fl/fl^*Mx1-Cre* and control mice were treated with pIpC as shown in Figure 1B. (A) CFC assay performed on total BM cells from *Cited2*^Δ/Δ^ and control mice. The graph shows the mean number of CFC colonies ± SD counted on day 10 (n = 3 per group). (B) CAFC assay. The graph shows the mean number of cobblestone areas ± SEM counted at week 5 (n = 3). (C) Competitive repopulation assay. CD45.2^+^ BM cells from *Cited2*^Δ/Δ^ or control mice were mixed with CD45.1^+^ WT competitor BM cells and transplanted into irradiated CD45.1^+^ WT recipients. After 16 weeks, the contribution of CD45.2^+^ cells was analyzed. Data are mean percentage of CD45.2^+^ cells in PB of recipient mice ± SD (n = 6 per group). (D) Frequencies of the BM LSK and Lin^−^Sca-1^−^c-Kit^+^ (LK) cells from *Cited2*^Δ/Δ^, *Cited2*^+/Δ^, and *Cited2*^fl/fl^ control mice. The data are representative of four independent experiments. (E) BM cells from untreated *Cited2*^fl/fl^*Mx1-Cre* and *Cited2*^fl/fl^ mice were mixed with CD45.1^+^ WT competitive BM and transplanted into irradiated recipients. Eight weeks after tansplantation, the mice were treated with five doses of pIpC. Five days after last pIpC administration, the percentage of test CD45.2^+^ cells was measured in BM LSK and LK compartments. Data are mean ± SD (n = 3). ^∗^p < 0.001; ^∗∗^p < 0.0002. (F) LTC-IC assay. Human CD34^+^ CB cells transduced with shRNA and control lentiviruses were cocultured with MS5 stromal cells. After 5 weeks, medium was replaced with complete methylcellulose. The graph shows the mean percentage of GFP^+^ LTC-IC colonies in cultures ± SD (n = 2) scored 2 weeks after adding methylcellulose.

Next, we addressed the impact of *Cited2* deletion on HSC and progenitor cell activity. In colony-forming cell (CFC) assays, *Cited2*^Δ/Δ^ BM cells failed to generate colonies in methylcellulose (Figure 3A). To evaluate HSC activity in vitro, we performed limiting dilution cobblestone area-forming cell (CAFC) assays and found that *Cited2*^Δ/Δ^ BM completely lacked CAFCs (Figure 3B). To assess HSC activity in vivo, we transplanted CD45.2^+^ BM cells from *Cited2*^Δ/Δ^ and *Cited2*^fl/fl^ control mice (with or without WT CD45.1^+^ BM competitors) into irradiated congenic CD45.1^+^ recipients. Without CD45.1^+^ BM competitors, *Cited2*^Δ/Δ^ BM cells did not rescue recipient mice from lethal irradiation (data not shown). Furthermore, CD45.2^+^ *Cited2*^Δ/Δ^ BM cells transplanted with CD45.1^+^ BM competitor cells did not contribute to multilineage hematopoiesis (Figure 3C). Immunophenotypic analysis of *Cited2*^Δ/Δ^ BM revealed a near complete loss of cells in the LSK compartment (Figure 3D) that contains LT-HSCs, ST-HSCs, and LMPPs. The frequency of Lin^−^Sca-1^−^c-Kit^+^ (LK) myeloid progenitor cells was also profoundly decreased in *Cited2*^Δ/Δ^ mice. To exclude the effects of pIpC-induced Cre-mediated toxicity on hematopoietic stem and progenitor cells (HSPCs), we compared the immunophenotypic and functional properties of HSPCs from *Cited2*^+/+^ *Mx1-Cre* and *Cited2*^fl/fl^ mice and found no apparent differences (Figures S2A–S2D). These data indicate that pIpC-induced Cre activity does not phenocopy *Cited2* deletion in HSPCs.

The rapid kinetics of HSC loss upon acute deletion of *Cited2* suggest a survival defect. To test this, we deleted *Cited2* in cultured LSK cells and demonstrated that the rate of apoptosis was markedly increased in *Cited2*^Δ/Δ^ cells, as compared to WT cells (Figure S2E). Thus, decreased survival of LSK cells underpins the multilineage BM failure observed in *Cited2*^Δ/Δ^ mice.

### *Cited2* Functions in a Cell-Autonomous Manner in HSCs

To independently examine whether loss of *Cited2*^Δ/Δ^ HSCs is caused by *Cited2* deletion specifically in the hematopoietic system, we mixed CD45.2^+^ BM cells from untreated *Cited2*^fl/fl^ *Mx1-Cre* or *Cited2*^fl/fl^ mice with CD45.1^+^ WT BM competitor cells and transplanted them into irradiated recipients. Eight weeks after transplantation, the mice received pIpC and five days after the last dose the percentage of the donor-derived CD45.2^+^ cells was analyzed in the BM. The percentage of CD45.2^+^ *Cited2*^Δ/Δ^ cells in LSK and LK compartments was significantly reduced compared to CD45.2^+^ *Cited2*^fl/fl^ cells (Figure 3E). These data indicate a cell-autonomous requirement for *Cited2* in HSC maintenance.

### *CITED2* Is a Regulator of Primitive Hematopoietic Cell Function in Human Cord Blood

The high evolutionary conservation of Cited2 in mammals (Bhattacharya et al., 1999) suggests a conserved role for Cited2 in HSC function. We generated a lentivirus expressing short-hairpin RNA (shRNA) targeting human *CITED2* (Figures S3A–S3D) and performed assays to enumerate LTC-ICs, the most primitive human progenitors assessable in vitro. CB CD34^+^ cells transduced with shRNA and control lentiviruses were cocultured on stromal cells. *CITED2* knockdown in CD34^+^ cells led to a severe reduction in cellularity over time, compared to CD34^+^ cells transduced with a control lentivirus (Figure S3E). Furthermore, *CITED2* knockdown in CD34^+^ cells strikingly impaired primitive hematopoietic cell activity, as judged by LTC-IC assays (Figure 3F). Thus, our data indicate that *CITED2* is a conserved regulator of primitive hematopoietic cell function in mammals. Furthermore, with this *Mx1-Cre*-independent model system, we corroborate the data obtained in our conditional mouse model.

### Intact *Ink4a/Arf* and *Trp53* Are Required for the Loss of *Cited2*^Δ/Δ^ HSCs

**Figure 4:**
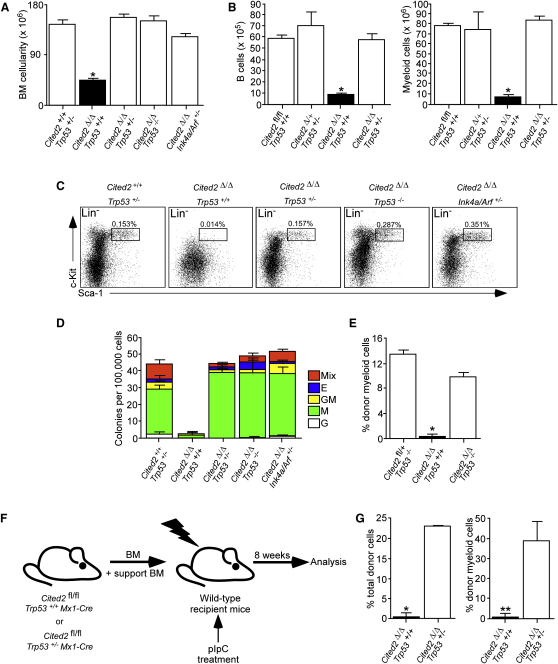
Genetic Deletion of *Trp53* or *Ink4a/Arf* Restores HSC Functions and Rescues Bone Marrow Failure in *Cited2*^Δ/Δ^ Mice Mice of indicated genotypes were treated with pIpC. (A) Total BM cellularity from two tibias and two femurs. The results are presented as mean number of cells ± SD (n = 3 per genotype). ^∗^p < 0.002 versus remaining genotypes. (B) Graphs show total number of BM CD19^+^B220^+^ cells (B cells) and Mac-1^+^Gr-1^+^ cells (myeloid cells) in two tibias and two femurs per mouse. Mean values ± SD (n = 4). ^∗^p < 0.005 versus remaining genotypes. (C) Frequencies of the BM LSK cells from mice of indicated genotypes. FACS plots are representative of three independent experiments. (D) CFC assay. Nucleated BM cells were plated in methylcellulose medium. Cultures were assessed on day 10 for granulocyte (CFC-G), macrophage (CFC-M), granulocyte-macrophage (CFC-GM), erythroid (E), and mixed (Mix) colony formation. The data are representative of three independent experiments and are shown as the mean ± SD (n = 2 mice per genotype). (E) Contribution of donor cells from *Cited2*^fl/+^*Trp53*^−/−^, *Cited2*^Δ/Δ^*Trp53*^+/+^, and *Cited2*^Δ/Δ^*Trp53*^−/−^ mice to the myeloid compartment of PB 16 weeks after transplantation. BM cells from mice of the indicated genotypes were mixed with support WT BM cells and transplanted into irradiated recipients. The graph shows the mean (±SD) percentage of CD45.2^+^ cells in myeloid compartment of recipient mice (n = 3 per group). ^∗^p < 0.0003 versus remaining genotypes. (F) Schematic of experimental design. (G) Contribution of donor cells of the indicated genotypes to PB. Percentage of lacZ^+^ donor cells was analyzed by flow cytometry in total PB mononuclear compartment and myeloid (Mac-1^+^Gr-1^+^) compartment of recipients (n = 5 per group). ^∗^p < 0.002; ^∗∗^p < 0.00008.

We previously showed that *Cited2* null MEFs senesce prematurely and have increased levels of p16^Ink4a^ and p19^Arf^ (Kranc et al., 2003), whereas ectopic expression of *Cited2* represses *p16^Ink4a^* and *p19^Arf^*, enhancing MEF proliferation. Deletion of *Ink4a/Arf* or *Trp53* (encoding p53, a downstream target of p19^Arf^), rescued defective proliferation in *Cited2*^−/−^ MEFs (Figure S4A; Kranc et al., 2003). *Ink4a/Arf* and *Trp53* are essential in maintaining HSC function (Akala et al., 2008), so we hypothesized their involvement in the loss of *Cited2*^Δ/Δ^ HSCs. Consistent with this, *Cited2* deletion in LSK cells resulted in an increased expression of p19^Arf^ and p53 proteins and a p53 target gene *Cdkn1a* (Figures S4B–S4D). Next, we generated *Cited2*^fl/fl^ *Mx1-Cre Trp53*^+/−^, *Cited2*^fl/fl^ *Mx1-Cre Trp53*^−/−^, *Cited2*^fl/fl^ *Mx1-Cre Ink4a/Arf* ^+/−^, and control mice and treated them with pIpC. Q-PCR confirmed that *Cited2* was not expressed in *Cited2*^Δ/Δ^ BM cells, regardless of *Ink4a/Arf* and *Trp53* status (Figure S4E). Deletion of one *Ink4a/Arf* allele or one or two alleles of *Trp53* restored total BM cellularity in *Cited2*^Δ/Δ^ mice to the levels observed in *Cited2*^fl/fl^ control mice (Figures 4A and 1D). Ablation of one allele of *Trp53* also rescued B cell and myeloid development in *Cited2*^Δ/Δ^ BM (Figure 4B). Furthermore, deletion of one allele of *Ink4a/Arf* or one or two alleles of *Trp53* restored BM *Cited2*^Δ/Δ^ LSK cells (Figure 4C). BM cells from *Cited2*^Δ/Δ^ *Trp53*^+/−^, *Cited2*^Δ/Δ^ *Trp53*^−/−^, and *Cited2*^Δ/Δ^ *Ink4a/Arf* ^+/−^, but not *Cited2*^Δ/Δ^, mice efficiently generated multilineage colonies in methylcellulose (Figure 4D). After confirming that *Cited2*^Δ/Δ^ *Trp53*^+/−^, *Cited2*^Δ/Δ^ *Trp53*^−/−^, and *Cited2*^Δ/Δ^ *Ink4a/Arf* ^+/−^ cells from primary colonies lacked *Cited2* expression, we demonstrated efficient generation of secondary colonies (data not shown).

To examine whether HSCs lacking both *Cited2* and *Trp53* have long-term repopulating capacity, we transplanted *Cited2*^Δ/Δ^ *Trp53*^+/+^, *Cited2*^Δ/Δ^ *Trp53*^−/−^, and *Cited2*^fl/+^ *Trp53*^−/−^ total BM cells (mixed with WT support BM cells) into irradiated recipients and analyzed peripheral blood (PB) 16 weeks after transplantation. *Cited2*^Δ/Δ^ *Trp53*^+/+^ BM cells failed to repopulate recipients (Figure 4E), whereas BM cells lacking both *Cited2* and *Trp53* repopulated recipients to a similar extent as those lacking *Trp53* with intact *Cited2*. To corroborate this, we transplanted BM cells from untreated *Cited2*^fl/fl^ *Mx1-Cre Trp53*^+/+^ and *Cited2*^fl/fl^ *Mx1-Cre Trp53*^+/−^ mice into irradiated recipients (Figure 4F). After reconstitution, the recipients were treated with pIpC and analyzed 8 weeks after administration of the last dose. We measured the percentage of donor cell chimerism in PB nucleated cells or myeloid cells of recipients by using lacZ as a marker of *Cited2*-deficient cells. Whereas *Cited2*^Δ/Δ^ *Trp53*^+/+^ cells failed to sustain hematopoiesis, those lacking *Cited2* and one allele of *Trp53* showed significant donor-derived contribution (Figure 4G). Together, these data provide genetic evidence that the loss of HSCs in *Cited2*^Δ/Δ^ mice is, at least in part, mediated by *Ink4a/Arf* and *Trp53*.

## Discussion

In this report, we investigate the requirement for *Cited2* in adult HSCs maintenance and committed hematopoietic lineages. By using an inducible conditional knockout approach in adult mice, we demonstrate that *Cited2* deletion results in an acute loss of HSCs, at least in part via apoptosis, subsequently causing multilineage BM failure. Specific deletion of *Cited2* within the hematopoietic system demonstrates a cell-autonomous requirement for *Cited2* in maintaining adult HSC integrity, whereas deleting *Cited2* in committed lymphoid and myeloid lineages has no impact. Furthermore, *CITED2* knockdown in human CD34^+^CB reveals a conserved requirement for *Cited2* in HSC maintenance. Together, our data provide evidence that *Cited2* functions in a cell-autonomous manner to maintain HSCs.

Genetic evidence indicates that the tumor suppressors *Ink4a/Arf* and *Trp53* regulate multiple HSC fate decisions (Akala et al., 2008; Liu et al., 2009; Oguro et al., 2006). One function of p19^Arf^ is to stabilize p53 (Pomerantz et al., 1998), and the activation of the p19^Arf^-p53 pathway results in loss of HSCs (Park et al., 2003). We showed that loss of *Cited2* increased p19^Arf^ and p53 expression in the LSK compartment. Based on this observation, we used a genetic rescue approach to test whether *Ink4a/Arf* and *Trp53* are required for loss of HSCs lacking *Cited2*. Our results demonstrated that deletion of *Ink4a/Arf* or *Trp53* restored functionality of HSCs lacking *Cited2*, implying that *Cited2* maintains HSCs, at least in part, via *Ink4a/Arf* and *Trp53*. These data support the postulate that deletion of *Cited2* in HSCs results in activation of the p19^ARF^-p53 pathway and thereby leads to their loss.

It is of interest to relate Cited2 to other critical regulators of HSC maintenance. *Cited2* is required for *Bmi-1* expression in MEFs (Kranc et al., 2003) and myeloid progenitors (Chen et al., 2007). *Bmi-1* maintains HSCs (Lessard and Sauvageau, 2003; Park et al., 2003) and directly represses *Ink4a/Arf* (Bracken et al., 2007), whereas deletion of *Ink4a/Arf* (Oguro et al., 2006) or *Trp53* (Akala et al., 2008) restores *Bmi-1*^−/−^ HSC function. Genetic evidence indicates distinct roles for *Bmi-1* and *Cited2* in HSC fate decisions. Whereas *Bmi-1* mediates HSC self-renewal, our results are compatible with a requirement for *Cited2* in HSC survival. In agreement with this, acute *Cited2* deletion in HSCs does not affect the expression of *Bmi-1* (data not shown), suggesting that downregulation of *Bmi-1* expression is not responsible for the loss of *Cited2*^Δ/Δ^ HSCs. However, this does not exclude the possibility that *Cited2* controls *Bmi-1* in other contexts in HSCs. Conditional deletion of *Cited2* generates a stem cell phenotype reminiscent of conditional inactivation of *Tel/Etv6* and *Mcl-1* (Hock et al., 2004; Opferman et al., 2005). Like *Tel/Etv6* (Hock et al., 2004), *Cited2* appears to be selectively required for HSC maintenance, but dispensable for mature lineages. Mcl-1, however, also plays critical roles in mature T and B cell survival (Opferman et al., 2003), revealing a broader spectrum of hematopoietic function than Cited2. Conditional deletion of *Apc* and combined deficiency of *c-Myc* and *N-Myc* (but not ablation of *N-Myc* alone) results in loss of HSCs (Laurenti et al., 2008; Qian et al., 2008). Although the expression of *Apc* and *c-Myc* is unaltered in *Cited2*-deficient HSCs, the expression of *N-myc* is decreased (data not shown). Although this observation alone does not explain the loss of *Cited2*-deficient HSCs, N-Myc may mediate some functions of Cited2 in HSCs. Finally, Cited2 binds p300 and its paralog CBP (Bhattacharya et al., 1999). Although *Cbp* is essential for adult HSC maintenance, *p300* appears dispensable for HSC maintenance but required for multilineage hematopoietic differentiation (Kung et al., 2000; Rebel et al., 2002). It will be of interest to clarify the roles of Cbp-Cited2 and p300-Cited2 interactions in adult HSC maintenance and hematopoiesis, and the relationship between Cited2 and other critical stem cell regulators remains an open question meriting future investigation.

In conclusion, we provide genetic evidence that *Cited2* is an essential and cell-autonomous regulator of adult mammalian HSC maintenance. Our data, together with the sufficiency of Cited2 to maintain undifferentiated embryonic stem cells (Pritsker et al., 2006), suggest that it is a critical master regulator of stem cell fate. Understanding Cited2 functions at the molecular level will offer insights into the similarities and differences in the transcriptional circuitry of embryonic and somatic stem cells.

## Experimental Procedures

### Mice

We backcrossed *Cited2*^fl/fl^ and *Cited2*^+/−^ mice (Bamforth et al., 2001; MacDonald et al., 2008) to C57BL/6J for ten generations to generate coisogenic mice. *Mx1-Cre*, *Cd19-Cre*, and *LysM-Cre* mice were purchased from the Jackson Laboratory. *Cd4-Cre* mice were purchased from Taconic. *Ink4a/Arf* ^+/−^ and *Trp53*^+/−^ mice were obtained from B. Hassan and M. van Lohuizen, respectively. All experiments on animals were performed under UK Home Office authorization.

### Administration of pIpC

8- to 12-week-old mice received five to six intraperitoneal injections of pIpC (GE Healthcare; 0.2–0.3 mg per dose) every alternate day. Deletion efficiency was determined by Q-PCR or lacZ expression analysis (via a FluoReporter lacZ Flow Cytometry Kit, Invitrogen).

### Murine CAFC Assay

Stromal layers were prepared from the BM of C57BL/6J mice, irradiated at 15 Gy, and subcultured in 96-well flat-bottom plates at a density of 2 × 10^4^ cells per well. After 1 to 7 days, cultures were seeded at 2-fold dilutions (2.9 × 10^5^−18,125 per well) of nucleated BM cells from each genotype. CAFCs were scored at week 5.

### CFC Assays

H4434 and M3434 media (StemCell Technologies) were used to enumerate human and mouse colony-forming cells, respectively. Two replicates were used per group in each experiment. Colonies were tallied at day 10–14.

### Q-PCR

RNA extraction and Q-PCR reactions were performed as previously described (Mansson et al., 2007). For specific TaqMan Assays-on-Demand probes used, see Supplemental Experimental Procedures. Reactions were run on an Applied Biosystems 7500 Fast Real-Time PCR System in normal mode for 50 cycles. All experiments were performed in triplicate. Differences in input cDNA were normalized with a combination of *Hprt*, *Gapdh*, *Actb, Ubc*, and *B2m* expression with qBase 1.3.5 software (http://medgen.ugent.be/qbase/).

### Lentiviral Transductions

*CITED2* shRNA was subcloned from the pLKO.1 puro vector (Open Biosystems) into the pLKO.1 GFP vector (gift from J. Larsson). Lentivirus production and transduction of human CD34^+^ CB cells are described in Supplemental Experimental Procedures.

### Human Long-Term Cultures on Stroma and LTC-IC Assays

CB CD34^+^ cells (StemCell Technologies) were isolated by MiniMACS (Miltenyi Biotec) selection. After transduction, 3 × 10^4^ cells were cultured on MS5 stromal cells in Long-Term Culture medium (see Supplemental Experimental Procedures). Cultures were demidepopulated weekly for analysis. LTC-IC numbers were enumerated by overlaying MS5 stromal cocultures at week 5 with H4434 medium, followed by counting colonies 2 weeks later.

### FACS

All samples were analyzed on a CyAn ADP flow cytometer (Dako). Sorts were performed on FACSAriaIIu (BD) or MoFlow (Dako) cell sorters. Antibodies are described in Supplemental Experimental Procedures.

### Competitive Repopulation Assay

CD45.2^+^ test donor BM cells were mixed with CD45.1^+^ competitor BM cells in a 1:1 ratio and injected intravenously into lethally irradiated (9 Gy) B6.SJL CD45.1^+^ recipients. The competitor cell number was 5 × 10^5^ cells in all experiments.

### Statistical Analysis

Statistical significance was determined via two-tailed Student's t tests assuming unequal variance.

## Supplemental Data

Document S1. Supplemental Experimental Procedures and Four Figures
