# Supplementary material for: Cited2 Is an Essential Regulator of Adult Hematopoietic Stem Cells
Source: Cell Stem Cell. 2009 Dec 4;5(6):659–65. doi: 10.1016/j.stem.2009.11.001 (PMC2828538; doi:10.1016/j.stem.2009.11.001)
Supplement: Document S1. Supplemental Experimental Procedures and Four Figures [file mmc1.pdf]

Cell Stem Cell, Volume *5*

Supplemental Data

Cited2 Is an Essential Regulator of Adult Hematopoietic Stem Cells

Kamil R. Kranc, Hein Schepers, Neil P. Rodrigues, Simon Bamforth, Ellen Villadsen, Helen Ferry, Tiphaine Bouriez-Jones, Mikael Sigvardsson, Shoumo Bhattacharya, Sten Eirik Jacobsen, and Tariq Enver

Supplemental Experimental Procedures:

Quantitative RT-PCR probes:

TaqMan Assays-on-Demand probes used were: *Cited2*: Mm00516121_m1; *Hprt*: Mm00446968_m1; *B2m*: Mm00437762_m1; *Gapdh*: Mm99999915_g1; *Actb*: Mm00607939_s1; *Ubc*: Mm01201237_m1; *Bmi-1*: Mm00776122_gH; *Mel18*: Mm00464756_m1; *Apc*: Mm00545877_m1; *c-Myc*: Mm00487803_m1; *N-Myc*: Mm00476449_m1; *Cdkn1a*: Mm00432448_m1; *CITED2*: Hs00366696_m1; *ACTB*: Hs99999903_m1; *B2M*: Hs00187842_m1; *GAPDH*: Hs99999905_m1; *HPRT1*: Hs99999909_m1.

Lentiviral transductions

The *CITED2* RNA hairpin was NdeI-SpeI subcloned from the pLKO.1 puro vector (Open Biosystems, Oligo ID: TRCN0000015654) into the pLKO.1 GFP vector (gift from J. Larsson, Lund University). The sequence of scrambled oligonucleotide used to generate shRNA was TTCTCCGAACGTGTCACGTT. $2.5x10^6$ 293T Human Embryonic Kidney cells were transfected with 3 μg pCMV Δ8.91, 0.7 μg VSV-G and 3 μg of either pLKO.1 scrambled shRNA (gift from J. Larsson, Lund University), pLKO.1 *CITED2* shRNA, *IRES-GFP* (control) or *CITED2-IRES-GFP* vectors. After 24 hours medium was changed to HPGM (Cambrex, Verviers, Belgium) and after 12 hours supernatant containing lentiviral particles was harvested and stored at -80ºC. Cord blood $CD34^+$ cells were

isolated with MiniMACS columns and subsequently cultured in HPGM supplemented with human c-KIT ligand, FLT3 ligand (both from Amgen, USA) and TPO (100 ng/ml each) for 16 hours at 37ºC and 5% CO_2. Cord blood $CD34^+$ cells were transduced in 2 consecutive rounds of 8 to 12 hours with lentiviral supernatant supplemented with c-Kit ligand/FLT3 ligand/TPO (100 ng/ml each) and Polybrene (4 µg/ml). Transduction efficiency was measured by FACS analysis and knock-down was assessed by Q-PCR.

FACS

Antibodies used for cell surface staining were CD19 (1D3), B220 (RA3-6B2), Gr-1 (RB6-8C5), CD11b/Mac-1 (M1/70), CD4 (H129.9), CD8a (53-6.7), SCA-1 (E13-161.7), c-KIT (2B8), CD45.2 (104), CD34 (RAM34), FLT3 (AZF10.1) (all from BD Pharmingen). Polyclonal goat-anti-rat Tricolor was purchased from Caltag.

Human cord blood cell culture conditions

Long Term Culture medium (αMEM supplemented with 12.5% FCS, 12.5% Horse serum (Gibco), 1% penicillin and streptomycin, 200 mM Glutamine, 57.2 µM β-mercaptoethanol (Sigma) and 1 µM hydrocortisone (StemCell Technologies)) was used to co-culture cord blood cells with MS5 stromal cells. Cultures were kept at 37ºC and 5% CO_2.

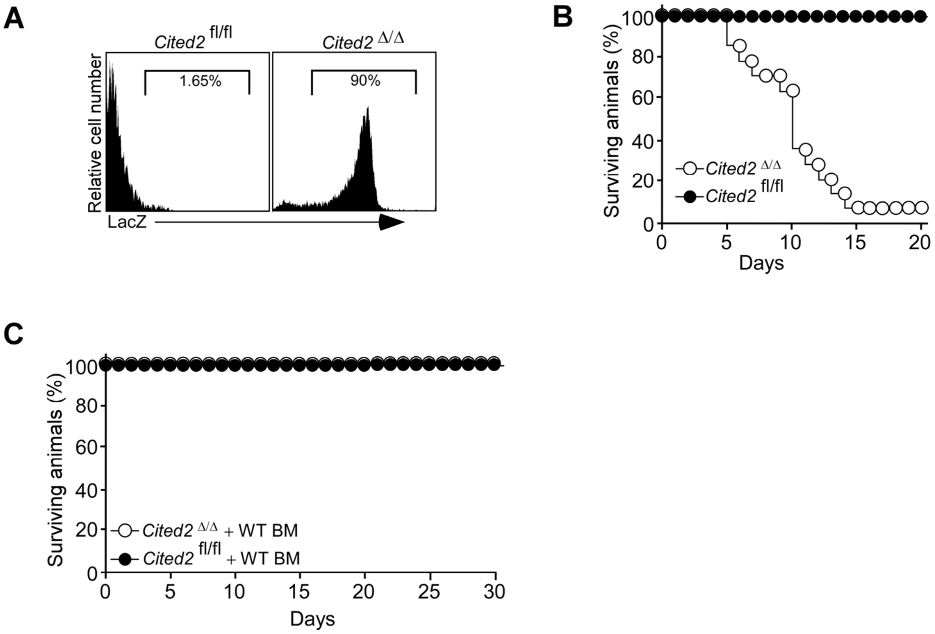

Figure S1. Deletion of *Cited2* in adult mice using *Mx1-Cre* results in premature lethality which can be rescued by wild-type bone marrow.

(A) LacZ expression in bone marrow cells from *Cited2*$^{\Delta/\Delta}$ and control mice. As Cre-mediated deletion of exon 2 brings the *lacZ* cassette under control of the endogenous *Cited2* promoter, the analysis of lacZ expression allows the confirmation of deletion of conditional alleles of *Cited2*.

(B) Kaplan-Meier survival curve for cohorts of 10 mice of each genotype injected with pIpC. Mice were treated with pIpC according to schematic in Figure 1B.

(C) Survival curve of *Cited2*$^{\Delta/\Delta}$ mice transplanted with wild-type bone marrow. Wild-type bone marrow cells were transplanted into lethally irradiated *Cited2*$^{fl/fl}$ *Mx1-Cre* and *Cited2*$^{fl/fl}$ control mice (n=9 for each genotype). 12 weeks after transplantation recipient mice were treated with 300 μg pIpC as indicated in Figure 1B. The graph shows the percentage of surviving mice.

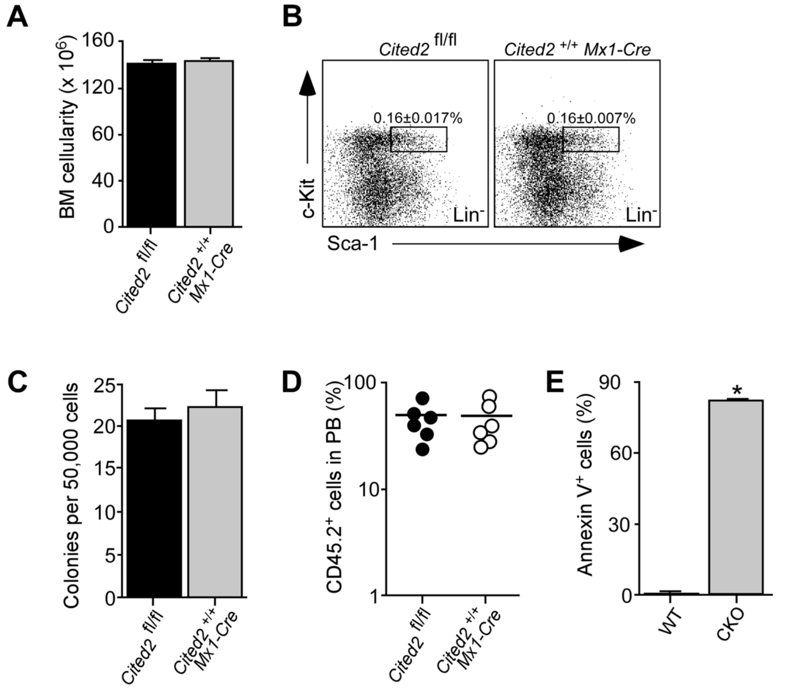

Figure S2. (A-D) Properties of stem and progenitor cells from *Cited2*$^{+/+}$ *Mx1-Cre* and *Cited2*$^{fl/fl}$ mice. (E) Defective survival of LSK cells lacking *Cited2*.

Cited2$^{+/+}$ *Mx1-Cre* and *Cited2*$^{fl/fl}$ mice were treated with pIpC according to the schematic in Figure 1B and BM was analysed 5 days after the last pIpC administration.

(A) BM cellularity in pIpC-treated *Cited2*$^{+/+}$ *Mx1-Cre* and *Cited2*$^{fl/fl}$ mice (n=3±SD).

(B) Immunophenotypic analysis of the BM LSK compartment in pIpC-treated *Cited2*$^{+/+}$ *Mx1-Cre* and *Cited2*$^{fl/fl}$ mice (n=3±SD).

(C) CFC assays with BM cells obtained from pIpC-treated *Cited2*$^{+/+}$ *Mx1-Cre* and *Cited2*$^{fl/fl}$ mice (n=3±SD).

(D) Competitive repopulation assay. BM cells from pIpC-treated *Cited2*$^{+/+}$ *Mx1-Cre* and *Cited2*$^{fl/fl}$ mice were mixed in a 1:1 ratio with competitor CD45.1$^+$ BM cells and transplanted into lethally irradiated CD45.1$^+$ recipients. Reconstitution was analysed 16 weeks after transplantation. Data are shown as percentage of CD45.2$^+$ cells in peripheral blood (n=6 mice per group).

(E) We bred *Cited2* conditional knockout mice with *Rosa26*$^{CreERT/+}$ mice, in which efficient deletion of floxed genes is induced by tamoxifen. We generated *Cited2*$^{fl/fl}$ *Rosa26*$^{CreERT/+}$ (CKO) and *Cited2*$^{+/+}$ Rosa26$^{CreERT/+}$ (WT) mice and sorted bone-marrow LSK populations from these mice. The cells were cultured in the presence of 1 μM tamoxifen and apoptosis was detected by Annexin-V staining. Graph shows percentage of Annexin-V$^+$ cells of indicated genotypes.

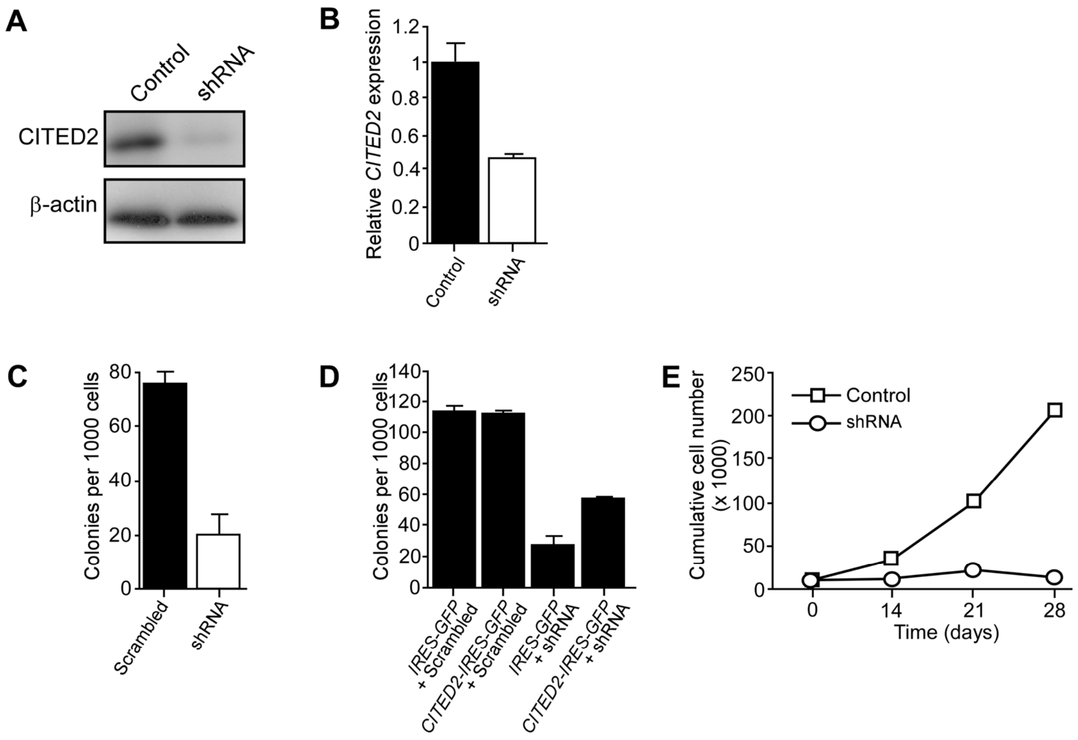

Figure S3. Human cord blood CD34$^+$ cells with *CITED2* knockdown fail to sustain hematopoiesis in vitro.

(A) 293T HEK cells were infected with control scrambled and shRNA lentiviruses. The efficiency of knockdown was determined by western blotting. *Top panel*: western blot of total cell lysates was probed with an anti-CITED2 monoclonal antibody (JA22, Abcam, Cambridge, UK). *Bottom panel*: western blot was re-probed with an anti-β-actin (C4, MP Biomedicals, UK) monoclonal antibody to confirm equal loading.

(B) Relative expression of *CITED2* mRNA in CD34$^+$ cord blood cells infected with lentiviruses expressing *CITED2* shRNA and a control lentivirus (scrambled shRNA). The data are presented as the mean ± SEM of triplicate assays in which *CITED2* expression was normalised to the expression of *GAPDH*.

(C) CFC assay. Human CD34$^+$ CB cells were infected with shRNA and control lentiviruses and cultured in methylcellulose for 2 weeks. The graph shows representative data (mean colony number ± SD) of 3 independent experiments performed in duplicate.

(D) CD34$^+$ cord blood cells were transduced with *CITED2-IRES-GFP* (expressing a human full-length CITED2 driven by human *EF1α* promoter) and control *IRES-GFP* lentiviruses. Transduced GFP$^+$ cells were then superinfected with shRNA and scrambled control lentiviruses and cultured in methylcellulose for two weeks. The graph shows the mean numbers of colonies (± SD) and is a representative of two independent experiments performed in duplicate.

(E) MS5 co-cultures of human cord blood CD34$^+$ cells transduced with shRNA and control lentiviruses. 30,000 cells were plated in MS5-precoated T25 culture flasks in 5 ml Long-Term Culture medium. Half of the cultures were harvested weekly and fresh medium was added to the culture. Cumulative cell numbers for a representative growth curve are shown (n=4).

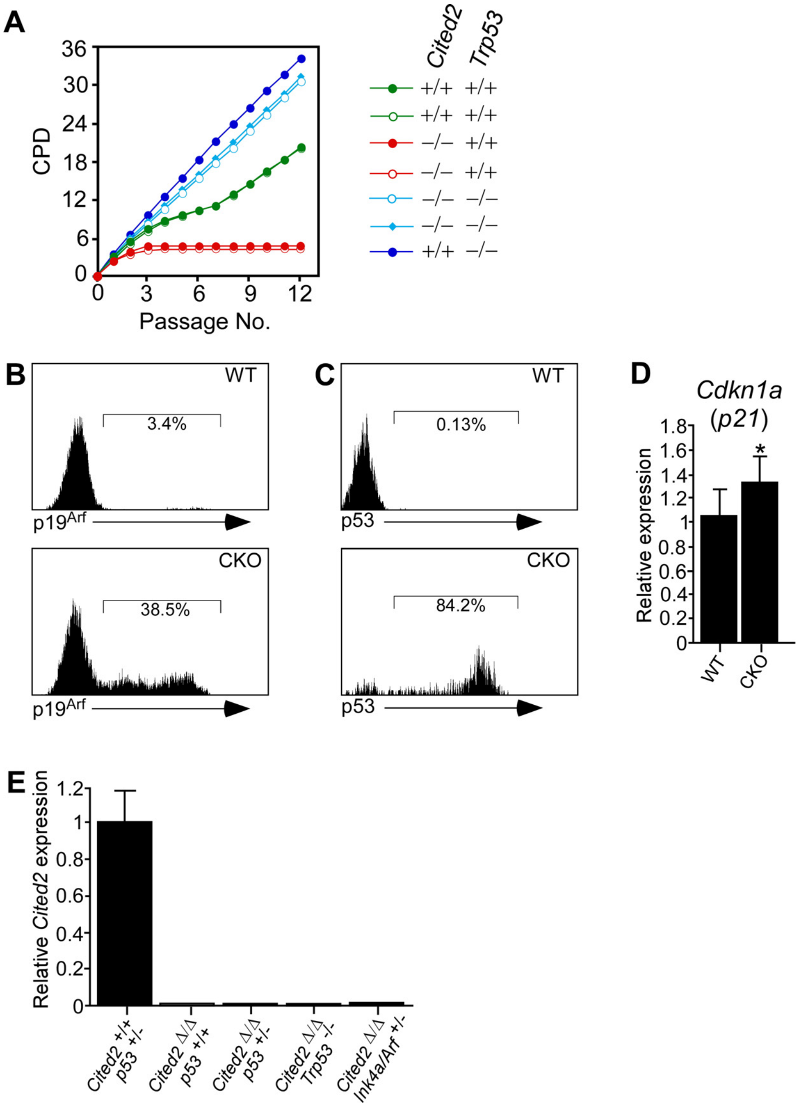

Figure S4. ***Cited2*** **functions genetically upstream of** ***Ink4a/Arf*** **and** ***Trp53*****.**

(A) MEFs obtained from embryos at 13.5 dpc were serially passaged according to the 3T3 protocol. Cell proliferation is shown as a plot of cumulative population doubling (CPD) versus passage number. Genotypes are indicated.

(B and C) BM LSK populations were sorted from *Cited2*$^{+/+}$ *Rosa26*$^{\mathrm{CreERT/+}}$ (WT) and *Cited2*$^{\mathrm{fl/fl}}$ *Rosa26*$^{\mathrm{CreERT/+}}$ (CKO) mice. The cells were cultured in StemSpan medium in the presence of SCF, FLT3 ligand and TPO (100 ng/ml each) and 0.5 μM tamoxifen for 2-3 days. Cells were fixed with 4% paraformaldehyde, permeabilized with 0.2% Triton-X100 and the expression of p19^{Arf} and p53 was detected using Ab80 (Abcam) and CM5 (Novocastra) antibodies, respectively. Data are representative of 2 experiments.

(B) Percentage of p19^{Arf}-expressing cells cultured in the presence of tamoxifen for 2 days.

(C) Percentage of p53-positive cells cultured with tamoxifen for 3 days.

(D) Expression of *Cdkn1a* in LSK cells lacking *Cited2*. LSK cells were sorted from *Cited2*$^{\mathrm{fl/fl}}$ (WT) and *Cited2*$^{\mathrm{fl/fl}}$ *Mx1-Cre* (CKO) mice. The cells were cultured in the presence of IFN-α for 36 hours to induce Mx1-Cre-mediated gene deletion. Q-PCR reactions were performed to determine the relative expression of *Cdkn1a* (n=3±SD, *p<0.005). *Cdkn1a* expression was normalised to the expression of *Hprt* and *Gapdh*.

(E) Relative expression of *Cited2* mRNA in total bone marrow samples obtained from mice used for experiments in Figure 4A, 4C and 4D. The data are presented as the mean

± SEM of triplicate assays in which *Cited2* expression was normalised to the expression of *Hprt*. Expression levels in *Cited2*$^{+/+}$ *Mx1-Cre Trp53*$^{+/-}$ mice were set to 1. Mouse genotypes are indicated.
